# Ranking metrics in gene set enrichment analysis: do they matter?

**DOI:** 10.1186/s12859-017-1674-0

**Published:** 2017-05-12

**Authors:** Joanna Zyla, Michal Marczyk, January Weiner, Joanna Polanska

**Affiliations:** 1 Data Mining Group, Institute of Automatic Control, Faculty of Automatic Control, Electronics and Computer Science, Silesian University of Technology, Akademicka 16, Gliwice, 44-100 Poland; 2 Max Planck Institute for Infection Biology, Charitéplatz 1, Berlin, 10117 Germany

**Keywords:** GSEA, Ranking metrics, Pathway analysis, Functional genomics

## Abstract

**Background:**

There exist many methods for describing the complex relation between changes of gene expression in molecular pathways or gene ontologies under different experimental conditions. Among them, Gene Set Enrichment Analysis seems to be one of the most commonly used (over 10,000 citations). An important parameter, which could affect the final result, is the choice of a metric for the ranking of genes. Applying a default ranking metric may lead to poor results.

**Methods and results:**

In this work 28 benchmark data sets were used to evaluate the sensitivity and false positive rate of gene set analysis for 16 different ranking metrics including new proposals. Furthermore, the robustness of the chosen methods to sample size was tested. Using k-means clustering algorithm a group of four metrics with the highest performance in terms of overall sensitivity, overall false positive rate and computational load was established i.e. absolute value of Moderated Welch Test statistic, Minimum Significant Difference, absolute value of Signal-To-Noise ratio and Baumgartner-Weiss-Schindler test statistic. In case of false positive rate estimation, all selected ranking metrics were robust with respect to sample size. In case of sensitivity, the absolute value of Moderated Welch Test statistic and absolute value of Signal-To-Noise ratio gave stable results, while Baumgartner-Weiss-Schindler and Minimum Significant Difference showed better results for larger sample size. Finally, the Gene Set Enrichment Analysis method with all tested ranking metrics was parallelised and implemented in MATLAB, and is available at https://github.com/ZAEDPolSl/MrGSEA.

**Conclusions:**

Choosing a ranking metric in Gene Set Enrichment Analysis has critical impact on results of pathway enrichment analysis. The absolute value of Moderated Welch Test has the best overall sensitivity and Minimum Significant Difference has the best overall specificity of gene set analysis. When the number of non-normally distributed genes is high, using Baumgartner-Weiss-Schindler test statistic gives better outcomes. Also, it finds more enriched pathways than other tested metrics, which may induce new biological discoveries.

**Electronic supplementary material:**

The online version of this article (doi:10.1186/s12859-017-1674-0) contains supplementary material, which is available to authorized users.

## Background

Ever since the high-throughput measuring techniques were introduced into molecular biology, methods for complex gene interaction analysis were developed (in parallel to methods for detecting differentially expressed genes). Throughout the years, three generations of gene set analysis methods were proposed. The first group is called Over-Representation Analysis (ORA) and was established in 1999 [1]. A wide array of tools belongs to this category, including simple ones, such as GOstat [2] and DAVID [3] or more sophisticated like RuleGO [4]. The main statistical assessment of a gene set significance in ORA is based on hypergeometric, *χ* ^2^ or Fisher exact test, which makes first generation methods simple and easy in implementation. However, there are two serious drawbacks of over-representation analysis: the information about the strength of feature differentiation is lost by binarisation (features in gene sets are represented only as differentially expressed genes or non-differentially expressed genes); assumption of gene expression independence in the hypergeometric test is not fulfilled in most of the cases. To overcome these problems, the second generation of gene set analysis methods was proposed in 2003 [5] and they are known as Functional Class Sorting (FCS). Those methods use information about all analysed genes and sort them according to some metric. The information from gene ranks is further transformed to pathway level (this process is specific to each algorithm) and statistical significance of each gene set is established. Nevertheless, gene sets are analysed independently (like in ORA), and direction of gene regulation according to biological knowledge is not incorporated. In literature several FCS methods were proposed, e.g. [6], CAMERA [7], PLAGE [8] and GSEA [5, 9]. In parallel the third generation of methods (Pathway Topology (PT)-based approaches) was developed in 2004 [10]. In their structure these methods are similar to FCS, but they use pathway topology to compute gene-level statistics. In this group methods like NetGSEA [11], CePa [12] or hybrid approach EnrichmentBrowser [13] were proposed. Even though the third generation methods seem to be the most consistent with the complexity of molecular level biology, they also have some limitations. The main one is that true pathway topology depends on cell cycle phase, cell types or specific conditions, but this information is nowadays rarely available. Additionally, they require much larger computational resources.

Despite drawbacks mentioned above, first and second generations methods are still commonly used. Among them, GSEA method seems to be the most popular with over 10,000 citations of original articles [9] in Google Scholar (over 1,000 citations only in 2016). Researchers apply this procedure to a variety of genomic studies, including large non-coding RNA [14], microRNA [15] or system biology of complex diseases [16, 17]. GSEA was constructed for analysis of gene expression data, however, there are extensions of the algorithm dedicated to deal with single nucleotide polymorphism data e.g. GSEA-SNP [18], MAGENTA [19] and i-GSEA4GWAS [20] or RNA-sequencing data e.g. SeqGSEA [21].

The first implementation of Gene Set Enrichment Analysis algorithm was created in Java by the authors of the original concept [22]. The biggest advantage of the application is a user friendly interface, a couple of different ranking methods and an access to gene set resources from Broad Institute. The same functionalities are provided in R package named GSEA-P-R. Recently, new implementations appeared. In rapidGSEA software suite [23] they proposed two tools for permutation-based GSEA using parallel computations on CUDA designated GPUs (cudaGSEA) or multi-core CPUs (ompGSEA). They introduced a simple gene ranking metric by calculating the local deviations. In both implementations it is not possible to use other gene ranking metrics.

The GSEA procedure is commonly used by the Java-based application [22], where the main parameter which needs to be set and can affect the final result is a choice of the ranking metric that measures the level of difference in gene expression between phenotypes. In the standard Java-based application basic metrics are implemented e.g.: signal-to-noise ratio (S2N), ratio of average expression from two classes (Ratio), T-test statistic (T-test), or the Pearson correlation coefficient for quantitative studies. However, researchers used also other gene ranking metrics e.g. logarithm transformed *p*-value from t-test [24], the Gaussian z-value corresponding to the one-sided *p*-value from the Welch t-test [25], Significance Analysis of Microarray [26]. New ranking metrics were used in combination with the pre-ranked GSEA procedure, where gene permutation is performed to obtain the enrichment score distribution. However, this type of permutation is not recommended, because it lose gene-gene correlation, so the phenotype sampling is more appropriate [9, 27].

Here, we present a complex comparison of multiple ranking metrics for GSEA, including ones implemented in standard Java application and novel metrics, that were successfully applied in feature selection of high-throughput data. To assess the effectiveness of tested ranking metrics, we propose two unique, statistically justified measures which are created by modification of those presented in [28]. The proposed measures are accompanied by computational time, and can be used in any other comparison study. Until now, there have been few studies where ranking metrics in Gene Set Enrichment Analysis were tested [29–31], however here we use a variety of ranks with powerful phenotype permutation, a large collection of data sets and statistical quality measures of gene set analysis. Finally, we have implemented GSEA method in MATLAB and named it MrGSEA (MATLAB metric GSEA - https://github.com/ZAEDPolSl/MrGSEA). The implementation includes all tested ranking metrics and leaves the possibility to implement new ranking metrics with the most powerful phenotype permutation. Additionally, the implementation has parallel computing capabilities. All of this can refine and extend the existing Java-based solution [22] or CUDA-based solution [23].

## Methods

### Data sets

The publicly available microarray data sets from two Bioconductor packages were used. In both collections, to each disease the target pathway from Kyoto Encyclopedia of Genes and Genomes (KEGG) [32] has been assigned, which provided the main information about efficiency of the tested gene ranking metric. The assignment of the target pathway to disease was performed based on KEGG Disease resource, where to each disease the leading pathways are pointed out. To each disease the pathway with the same name as a disease was chosen as a target pathway, e.g. when a subject of the study was a renal cancer, the target pathway is hsa:05211 termed Renal cell carcinoma. The first data collection is available in KEGGdzPathwaysGEO package [6] consisting of 24 microarray data sets, while second one is available in KEGGandMetacoreDzPathwaysGEO package [28] consisting of 18 microarray data sets. From both collections the data sets with paired study design and with Metacore pathways identification were removed. Additionally, data sets of clear cell renal cell carcinoma (ccRCC) previously used in [33], were downloaded from Gene Expression Omnibus [34] (IDs: GSE6344, GSE15641, GSE14994, GSE11024) and included into the analysis. The duplicates of probe set assignments to genes were removed according to the following procedures: for KEGGdzPathwaysGEO by keeping the probe set with the smallest *p*-value, and for KEGGandMetacoreDzPathwaysGEO and ccRCC data sets by keeping the probe set with the highest average expression across all samples [6]. For every data set, the proportion of non-normally distributed genes was found by Lilliefors test. Detailed description of the 28 data sets used is shown in Table 1. As a gene set collection KEGG pathways were used via KEGGREST package (updated on 11/2015) consisting of 299 different pathways.

**Table 1:** General information about used microarray data sets

GEO	Target KEGG ID	Disease/KEGG pathway name	Tissue	Sample Size (Control+Case)
GSE1145	hsa:05414	Dilated cardiomyopathy	Left Ventricle	26 (11+15)
GSE14924_CD4	hsa:05221	Acute myeloid leukemia	CD4 T cells	20 (10+10)
GSE14924_CD8	hsa:05221	Acute myeloid leukemia	CD8 T cells	21 (11+10)
GSE16759	hsa:05010	Alzheimer’s disease	Parietal lobe	8 (4+4)
GSE24739_G0	hsa:05220	Chronic myeloid leukemia	Peripheral blood	12 (4+8)
GSE24739_G1	hsa:05220	Chronic myeloid leukemia	Peripheral blood	12 (4+8)
GSE32676	hsa:05212	Pancreatic cancer	Pancreas	32 (7+25)
GSE4183	hsa:05210	Colorectal cancer	Colon	23 (8+15)
GSE1297	hsa:05010	Alzheimer’s disease	Hipopocampal CA1	16 (9+7)
GSE14762	hsa:05211	Renal Cancer	Kidney	21 (12+9)
GSE19188	hsa:05223	Non-small cell lung cancer	Lung	153 (62+91)
GSE19728	hsa:05214	Glioma	Brain	21 (4+17)
GSE20153	hsa:05012	Parkinson’s disease	Lymphoblasts	16 (8+8)
GSE20291	hsa:05012	Parkinson’s disease	Ppstmortem brain putmen	33 (19+14)
GSE21354	hsa:05214	Glioma	Brain, Spine	17 (4+13)
GSE3585	hsa:05414	Dilated cardiomyopathy	Subendocardial left ventricle	12 (5+7)
GSE4107	hsa:05210	Colorectal cancer	Mucosa	22 (10+12)
GSE5281_EC	hsa:05010	Alzheimer’s disease	Entorhinal cortex	21 (12+9)
GSE5281_HIP	hsa:05010	Alzheimer’s disease	Hippocampus	23 (13+10)
GSE5281_VCX	hsa:05010	Alzheimer’s disease	Primary visual cortex	31 (12+19)
GSE781	hsa:05211	Renal Cancer	Kidney	17 (5+12)
GSE8762	hsa:05016	Huntington’s disease	Lymphocytes	22 (10+12)
GSE9348	hsa:05210	Colorectal cancer	Colon	82 (12+70)
GSE9476	hsa:05221	Acute myeloid leukemia	Peripheral Blood	63 (37+26)
GSE6344	hsa:05211	Renal Cancer	Kidney	20 (9+11)
GSE15641	hsa:05211	Renal Cancer	Kidney	55 (23+32)
GSE14994	hsa:05211	Renal Cancer	Kidney	30 (8+22)
GSE11024	hsa:05211	Renal Cancer	Kidney	22 (12+10)

### Gene set enrichment analysis method

The Gene Set Enrichment Analysis method proposed by Subramanian et al. [9] remains one of the most popular method used for testing possible dis-regulations in pathways (gene sets) due to differences in expression of genes between analysed experimental conditions. It has been categorised as a second-generation method, competitive with sample randomisation (more details on the tested hypothesis are available in [27]). The general idea of GSEA method is to test whether the distribution of genes (according to an established ranking metric) in the gene set differs from a uniform distribution, using a weighted Kolmogorov-Smirnov test statistic. To establish interesting gene sets the Enrichment Score is calculated as a maximum deviation from zero between hits of genes *g* into gene set *S* marked as *P*_*hit*_ (Eq. 1) and genes *g* outside gene set *S* marked as *P*_*miss*_ (Eq. 2):

1 $$  P_{hit}(S,i)= \sum\limits_{\begin{array}{c} g_{j} \in S \\j \leq i \end{array} } \frac{|r_{j}|}{N_{R}}  $$

2 $$  P_{miss}(S,i)= \sum_{\begin{array}{c} g_{j} \notin S \\j \leq i \end{array} } \frac{1}{(N-N_{H})}  $$

where *N*_*R*_ is a sum of absolute values of ranking metrics for all genes in gene set *S*, *N*_*H*_ is a gene set size, *N* is a total number of analysed genes and *r* is a value of ranking metric, representing how strong is the difference in gene expression between experimental groups. The *i* and *j* are indicators of the position in the sorted list of gene ranks. The significance of an observed enrichment score is assessed by a permutation test. In GSEA method two types of permutations can be performed: by sample or by gene labels. Since only the sample permutation type allows to keep gene correlation structure, which is recommended [27], only this approach is considered in the presented work. Finally, to adjust estimated enrichment score for variation in gene set size, the normalised enrichment score is calculated. In this study, *p*-value of normalised enrichment score is used as a measure of pathway enrichment.

### Ranking metrics

We compared 16 ranking metrics divided into two groups. Detailed formulae for all tested metrics are presented in Table 2. The first group consists of metrics available in standard GSEA Java-based application [22]: signal-to-noise ratio (S2N; the default measure in GSEA), absolute value of signal-to-noise ratio (|S2N|), difference of expression means between classes (Difference), ratio of expression means of two classes (Ratio), log_2_ of Ratio (log_2_(Ratio)), and T-test statistic (T-test). The second group consists of ranking metrics originating from the field of feature selection and frequently applied for discovery of differentially expressed genes in high-throughput biological experiments. First two metrics are based on Moderated Welch Test statistic (MWT and its absolute value, |MWT|), calculated using weighted pooled and unpooled standard errors in the t-test procedure and adjusted by estimation of the gene-level variance across genes [35]. Next two ranking metrics use non-parametric test statistics: the Sum of Ranks (SoR) and Baumgartner-Weiss-Schindler test statistic (BWS) [36]. Both have been used in GSEA before [31]. In contrast to other methods, these metrics make no assumption about the distribution of gene expression data. The SoR is based on the sum of ranks for genes belonging to a particular experimental class. The BWS test is based on the squared value of the difference between two empirical distribution functions weighted by the respective variance and approximated by average of B statistics for each class. Neuhäuser showed that BWS gives a more accurate Type I error control and more power compared to the Wilcoxon test [37]. Two further metrics are derived from ReliefF algorithm, which for each gene assess a weight (from 1 as the best to -1 as the worst). The weight represents the best separation between classes based on nearest neighbor distance estimation [38]. To each weight, the tied rank is assigned as a second ReliefF-based metric (ReliefF ranked). Also, the weighted average difference method (WAD) and its absolute value (|WAD|) were included. According to the authors, WAD gives better sensitivity and specificity in identifying differentially expressed genes and more stable top-rank genes list compared to standard mean difference or fold change [39]. Second to last of tested ranking metric is the fold change rank ordering statistics (FCROS), which is based on a truncated mean calculated from the matrix of fold changes from pairwise comparison between sample groups [40]. Finally, we used the Minimum Significant Difference (MSD) [41] that is defined as the signed distance of the confidence interval (CI) of the logarithm of fold change (logFC) estimate from no change (zero). This can be interpreted as the most pessimistic estimate of logFC which is still within the 95% CI. A value of MSD metric shows that in 95% of the cases the log fold change will have at least this magnitude. Negative value of MSD indicates that logFC of zero is within the CI. Although, our implementation of MSD is parametric, in general the calculation of CI can be achieved in non-parametric framework.

**Table 2:** Description of ranking metrics sorted from the most parametric, through non-parametric to data mining methods

Metrics	Description	Comments	Ref.
T-test	$\frac {\overline {x_{1}} - \overline {x_{2}} }{\sqrt {\frac {{s_{1}^{2}}}{n_{1}}+ \frac {{s_{2}^{2}}}{n_{2}}}} $		[9]
MWT	$\frac {\overline {x_{1}} - \overline {x_{2}} }{se_{m}}$; $ {se_{m}^{2}}=\frac {d_{0}{s_{0}^{2}+d_{w}{s_{w}^{2}}}}{d_{0}+d_{w}}$	and absolute value	[35]
MSD	$\left \{\begin {array}{ll} {CI}_{left} & log(FC)>0 \\ -{CI}_{right} & log(FC)<0 \end {array}\right.$		[41]
S2N	$\frac {\overline {x_{1}} - \overline {x_{2}} }{s_{1}+s_{2}}$	and absolute value	[9]
WAD	*AD*∗*w*; $AD=\overline {x_{1}} - \overline {x_{2}} $	and absolute value	[39]
	$w=\frac {\overline {x}-min}{max-min} $; $\overline {x}=\frac {\overline {x_{1}} + \overline {x_{2}} }{2} $		
Difference	$\overline {x_{1}} - \overline {x_{2}}$		[9]
Ratio	$\frac {\overline {x_{1}}}{ \overline {x_{2}}}$	and log_2_	[9]
FCROS	${Mean}_{(truncated,10\%)}\left |\begin {array}{ccc}{FC}_{1,1}&...&{FC}_{1,k}\\.&&.\\.&...&.\\.&&.\\FC_{N,1}&...&{FC}_{N,k}\end {array}\right | $		[40]
	k - pairwise comparison; FC - fold change, N - no. of genes		
SoR	$\sum \limits _{i=1}^{N_{1}}R_{i}$ ; *N*_1_ - size of group 1; *R* - ranks of elements from group 1		[31]
BWS	$\frac {B_{1}+B_{2}}{2}$; $ B_{1}=\frac {1}{n_{1}}\sum \limits _{j=1}^{n_{1}}\frac {\left (R_{1}^{j}-\frac {n_{2}+n_{1}}{n_{1}}j\right)^{2}}{\frac {j}{(n_{1}+1)} \left (1-\frac {j}{(n_{1}+1)}\right) \left (\frac {n_{2}(n_{2}+n_{1})}{n_{1}}\right) }$		[31, 36]
	$B_{2}=\frac {1}{n_{2}}\sum \limits _{i=1}^{n_{2}}\frac {\left (R_{2}^{i}-\frac {n_{2}+n_{1}}{n_{2}}i\right)^{2}}{\frac {i}{\left (n_{2}+1\right)} \left (1-\frac {i}{(n_{2}+1)}\right) \left (\frac {n_{1}(n_{2}+n_{1})}{n_{2}}\right) }$		
ReliefF	$W-\frac {\sum _{k=1}^{K}D(x,h_{k})}{tK}+\sum \limits _{c\neq class(x)}^{} \frac {P(c)}{1-P(class(x))}\frac {\sum _{k=1}^{K}D(x,m_{k})}{tK}$	and tied rank	[38]

### Implementation

The Gene Set Enrichment Analysis method was implemented using 64-bit MATLAB R2016a programming environment. All ranking metrics tested in the publication are available. There is a possibility to use the external ranking metric method by applying an intrinsic MATLAB function. For every ranking metric the software calculates the *p*-value corresponding to a difference in expression between phenotypes by use of permutation test. The Enrichment Score distribution can be estimated by permutation of gene labels or phenotypes. Additionally, there is a function to create a custom gene set database from an Excel file containing genes grouped into gene sets. Results of the enrichment analysis are stored as MATLAB variables, in Excel files and as PNG images.

The algorithm was parallelised using the idea of replicated workers. Due to the nature of the GSEA method, each thread calculates the enrichment score and its distribution for different gene sets independently. Since the number of genes in each gene set is different, which cause divergent computational time, this solution provides the fastest results. The number of tasks performed in parallel depends on the number of available processor cores and MATLAB software license (maximum 12 threads with Parallel Computing Toolbox). The source code with an example data set and demonstration script can be freely downloaded from https://github.com/ZAEDPolSl/MrGSEA[42].

### Experimental design

The computational experiment is based on the collection of 28 microarray data sets, where to each of them a target pathway is referred. Sixteen GSEA ranking metrics were tested by two scores: i) surrogate sensitivity – a *p*-value of normalised enrichment score statistic for target pathway (the smaller, the better) (see [28] S1 Note) and ii) false positive rate (FPR) – the percent of false positives found at 5% significance level (the closer to 5%, the better). To assess FPR, the original phenotypes of each data set were permuted creating 50 independent data collections. To estimate the overall sensitivity of a given ranking metric, the conservative estimator $\hat \pi _{0}$ from Storey’s method for multiple testing was used [43]. The $\hat \pi _{0}$ represents a proportion of truly null tests with the expectation that all *p*-values will follow uniform distribution, where 1-$\hat \pi _{0}$ is the proportion of truly alternative tests. To estimate the overall FPR of the ranking metric, the absolute deviation of mean FPR (observed level) from 5% (expected level) was taken. Additionally, the computational load of each ranking metric was evaluated. Obtained scores (overall sensitivity, overall FPR, computational load) were normalised to one scale, and the k-means clustering procedure with Euclidean distance was used to divide ranking metrics due to total performance in gene sets enrichment. The number of clusters in k-means algorithm was set with the use of Dunn index [44]. Finally, for the best ranking metrics, the robustness to sample size was tested in the following scenario: the largest data set (GSE19188) was randomly divided 30 times into different sample size collections (10, 20, 30, 40, 60, 80 and 100) in a stratified manner. For each scenario, the surrogate sensitivity and FPR of gene set enrichment for a given method was calculated.

## Results and discussion

### Overall sensitivity and FPR of gene set analysis

**Fig. 1:**
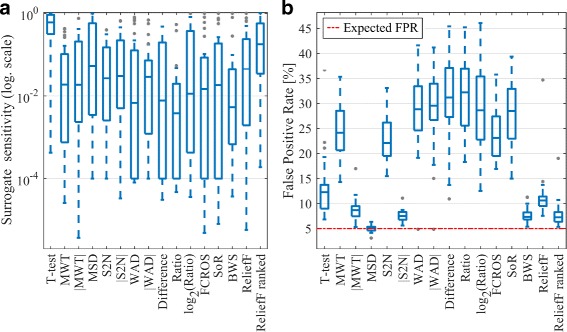
Boxplots of surrogate sensitivity and FPR of gene set analysis. Panel **a** represents the distribution of target pathways enrichment *p*-value to each metric presented in logarithmic scale - the lower the better; Panel **b** represents the results of FPR estimation, where the red line represents the expected outcome - the closer to 5% the better

For each of the 28 data sets the GSEA method, with 1000 phenotype permutations and 16 different ranking metrics, was performed. The *p*-values of target pathways, which represent surrogate sensitivity, are shown in Fig. 1 a. The average percent of significantly enriched gene sets, which represents FPR, is presented in Fig. 1 b. In all figures ranking metrics are sorted from the most parametric statistics, through non-parametric to data mining approaches. As can be seen in Fig. 1 a, we can distinguish two ranking metrics with the lowest median of surrogate sensitivity and relatively small spread of distribution, i.e. Ratio and BWS. The worst results are observed for T-test statistic and ReliefF ranked metric. In case of FPR, the group of seven metrics with a low value is observed: T-test, |MWT|, MSD, |S2N|, BWS, ReliefF and ReliefF ranked. To find the overall performance of ranking metric, expected outcomes for both scores were estimated: the 1-$\hat \pi _{0}$ estimator from Storey method [43] for overall sensitivity, and absolute value of difference between average FPR and expected one for overall FPR (Table 3). Ideally, for introduced definitions a ranking metric should have high value of overall sensitivity and low value of overall FPR. This approach highlights four ranking metrics: |MWT|, |S2N|, BWS and ReliefF. The Ratio metric has very good overall sensitivity, at the expense of poor FPR estimation, while opposite is observed for the ReliefF ranked metric and MSD. Additionally, detailed results of surrogate sensitivity and FPR for each ranking metric on every data set are presented in Additional file 1.

**Table 3:** Results of overall sensitivity, false positive rate and average evaluation time for all ranking metrics

Rank metric	$1-\hat \pi _{0}$	$ | \overline {FPR} - \widehat {FPR} |$	Average evaluation time [s]
T-test	0.066	8.162	118.363
MWT	0.928	19.634	191.944
|MWT|	0.998	3.665	191.944
MSD	0.559	0.008	117.627
S2N	0.926	17.932	115.090
|S2N|	0.981	2.542	115.090
WAD	0.992	23.482	112.534
|WAD|	0.994	23.971	112.534
Difference	0.985	25.212	111.920
Ratio	0.997	26.429	121.228
log_2_(Ratio)	0.824	23.337	120.820
FCROS	0.758	19.228	324.413
SoR	0.756	23.087	264.746
BWS	0.900	2.696	289.215
ReliefF	0.840	6.394	912.471
ReliefF ranked	0.548	2.852	912.471

Overall sensitivity is defined as 1 - estimator from Storey’s method (the higher, the better). Overall false positive rate is defined as an absolute value of the difference between observed and expected false positive rate (the lower, the better)

### Establishing the best ranking metrics

**Fig. 2:**
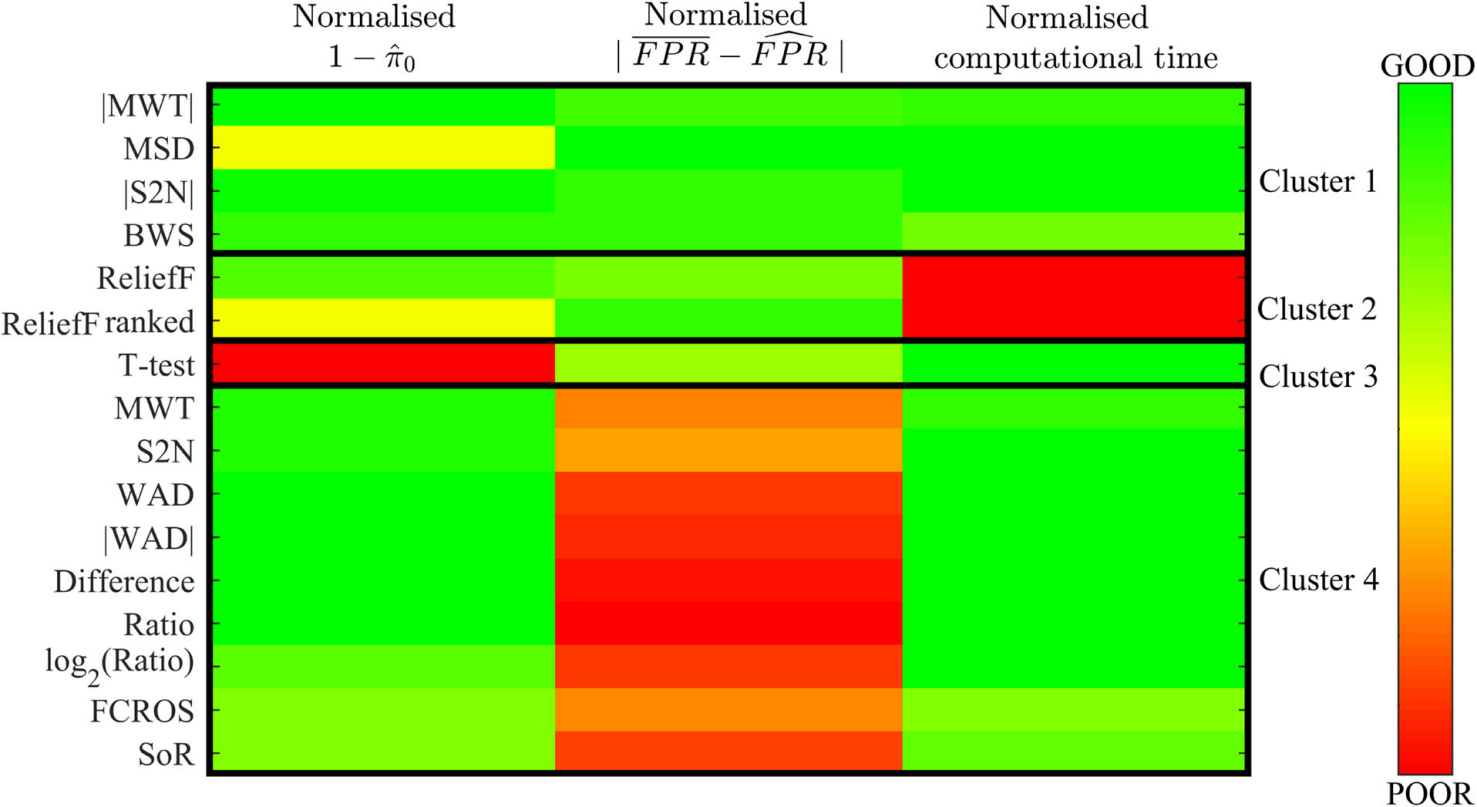
Results of k-means cluster analysis based on three performance criteria. Results highlighted with *green colour* show good performance, *red colour* represents poor performance and *yellow colour* represents medium performance

**Fig. 3:**
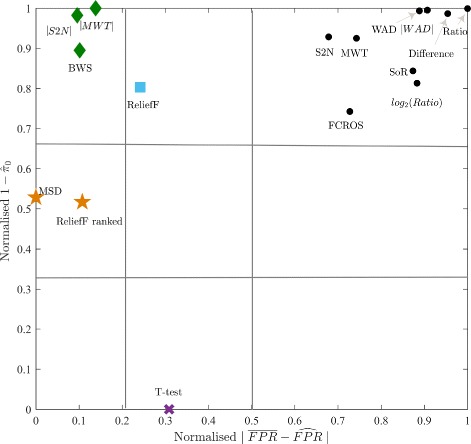
Results of k-means cluster analysis based on two performance criteria. The best results have those metrics, where FPR estimation is closest to 0, and sensitivity estimation (1-$\hat \pi _{0}$) is closest to 1

For all ranking metrics, the computational load was calculated as an important measure of practical application of given method (detailed evaluation for each metric and data set is included in Additional file 1). To find metrics which have the best overall sensitivity, overall FPR estimation and low computational cost, the k-means clustering approach with Euclidean distance was used. The estimators of each score were normalised to range [0, 1] to avoid favoring a single score. The Dunn index indicated four clusters as an optimal solution of clustering. Results of using k-means with 4 clusters are presented in Fig. 2. The most relevant clusters are the ones where computational load and overall FPR are low, and overall sensitivity is high. These three conditions are fulfilled for a cluster 1 (Fig. 2) with the metrics: |MWT|, MSD, |S2N| and BWS - green colour superiority. Out of those four metrics the |MWT| had the highest overall sensitivity, while MSD showed the lowest overall FPR and low computational load on the tested data collection. In this group only |S2N| is available in the original GSEA Java-based implementation [22], added as a consequence of results obtained in [45]. For other clusters showed in Fig. 2, we can observe a poor value for at least one of the tested measures (red colour). Additionally, the k-means procedure was applied only for overall sensitivity and overall FPR estimators to show another group with the best ranking metrics, when the computational load does not matter (Fig. 3). As can be observed the |MWT|, |S2N| and BWS are located in a cluster with the best outcomes, however this time k-means procedure also distinguished two other interesting clusters with weaker scores. The first cluster includes ReliefF metric which has medium FPR estimation and good overall sensitivity; the second cluster includes MSD and ReliefF ranked metrics, with low overall sensitivity but good FPR estimation.

### Robustness to sample size

**Fig. 4:**
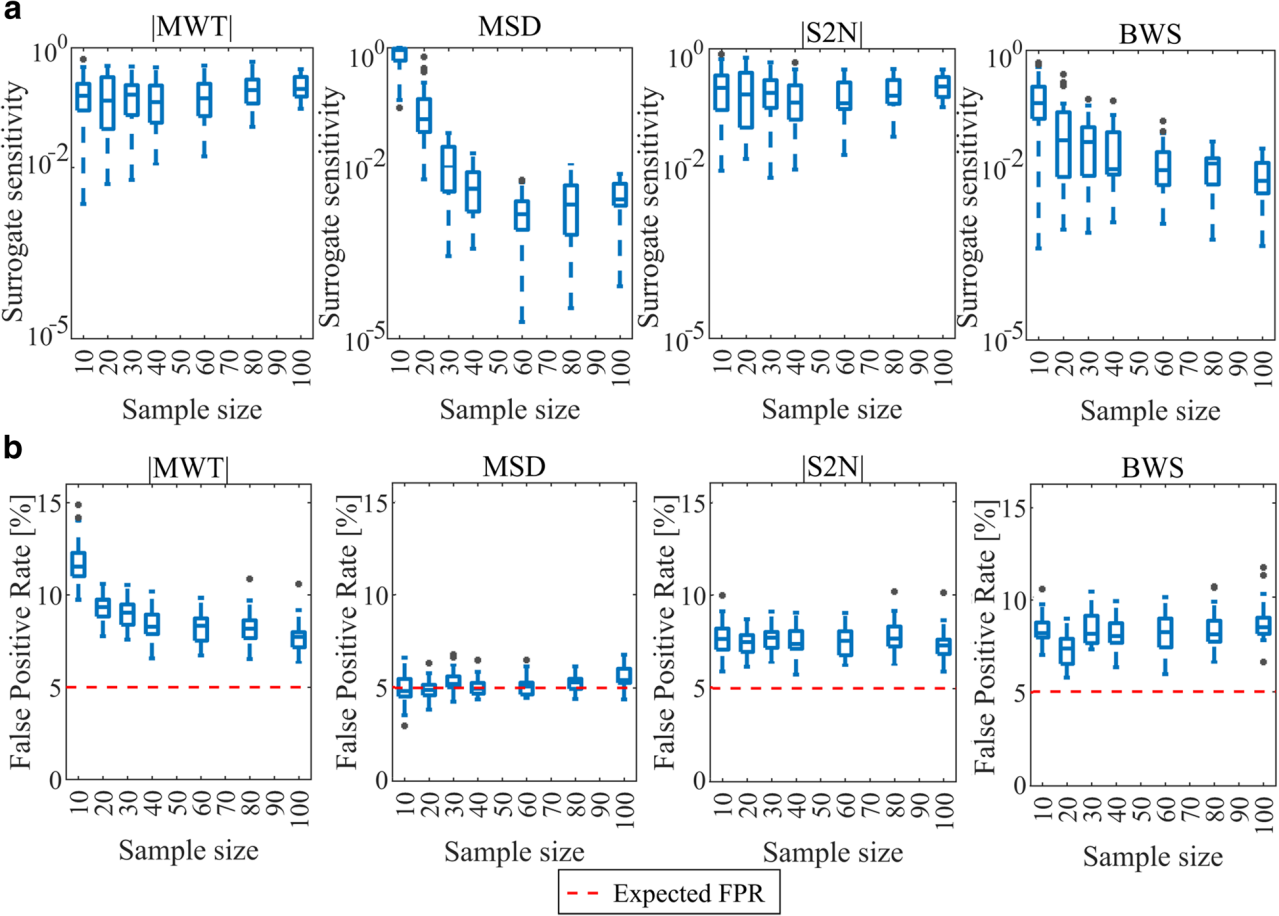
Robustness of ranking metrics to sample size. Panel **a** represents surrogate sensitivity assessment of four best metrics for different sample size. Panel **b** represents FPR estimates under tested sample size

The four best ranking metrics (|MWT|, MSD, |S2N| and BWS) were tested for robustness of pathway enrichment analysis with respect to number of samples in the analysed data set. Both surrogate sensitivity and FPR were assessed for each metric and different sample size (Fig. 4). Two metrics, |MWT| and |S2N| have similar levels of surrogate sensitivity, independent of sample size. BWS and MSD metrics show better results for larger sample sizes. In case of BWS it is related with the method of empirical estimation of B statistic. In case of MSD, it is caused by the weakness of standard error estimation for calculating the logFC CI. In case of FPR, it may be observed that it is constant for all metrics and sample sizes. The only exception is observed for |MWT| method, where for extremely low sample size the obtained FPR is higher. Analysis of robustness of the ranking metrics to sample size revealed that |MWT| and |S2N| give stable results, but for larger experiments obtained surrogate sensitivity is worse than for BWS and MSD.

### Precision in pathway enrichment

**Fig. 5:**
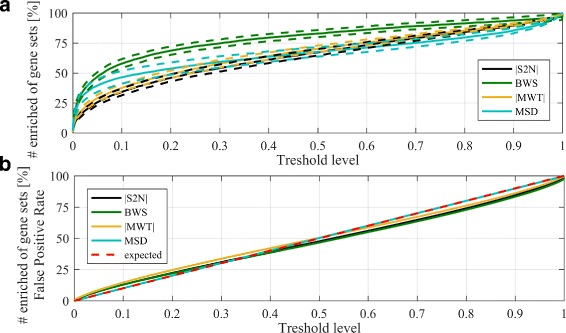
Results of detecting significant gene sets across various thresholds. Panel **a** represents percentage of significantly enriched pathways. *Solid lines* represent average value across analysed data sets whereas dashed lines represent its confidence intervals. Panel **b** represents percentage of significantly enriched pathways in experiment design dedicated to FPR evaluation. *Red dashed line* represents the expected outcome

Finally, the percentage of enriched pathways at different significance levels was determined. Figure 5 a, shows average percent of discovered pathways for tested data collection with 95% confidence intervals, while Fig. 5 b represents average level of enriched pathways after random permutation of sample labels (false positive estimation). As can be seen the Baumgartner-Weiss-Schindler test ranking metric gives the highest rate of statistically significant gene sets at different threshold levels, keeping the false positive results at the expected level. Results obtained for |S2N|, |MWT| and MSD are very similar and do not differ at 0.05 significance level between each other. For these three methods the expected level of number of false positives is also preserved. Those results show that BWS ranking metric not only finds expected target pathways with low *p*-values, but can detect more dis-regulated pathways in comparison to other metrics, with accepted number of false positive findings. Further research is needed to confirm the link between additional significant gene sets found by BWS and phenotypes under study in 28 data sets collection.

### Selected attributes of the best ranking metrics

Three of four best ranking metrics (|S2N|, MWT and MSD in the current implementation) are calculated using arithmetic mean and standard deviation. As these are parametric statistics, two assumptions are made: normality of data distribution and absence of outliers. In case of gene expression obtained using high-throughput biological techniques the assumption about normality of distributions is often not met and outliers are frequently present. Thus, it may lead to reduced power of pathway enrichment analysis. The data collection used in this study is characterised by not strongly skewed distributions of gene expression (see Additional file 2), so the described disadvantage of parametric methods is not evident. At 0.05 significance level, about 25% genes, on average, has a non-normal distribution, but when we set the significance level to 0.01 this number is reduced to 11%. Notably, whether MSD depends on parametric assumptions it depends on the specific implementation. On the other hand, the BWS test statistic has no assumption about the distribution of data, which allows to use this metric for every kind of biological data. In a group of analysed data sets the proportion of non-normally distributed genes is significantly correlated with gain in overall sensitivity obtained when using BWS comparing to |MWT| and |S2N| (*p*-value=1.16E-4 and 1.03E-4, respectively). No significant overall sensitivity gain is observed for comparison of BWS to MSD (*p*-value=4.56E-1). These findings are similar to the one obtained by Neuhäuser and Senske [37]. They also demonstrate that in case of symmetrical distributions BWS has similar power to parametric tests.

The GSEA method allows to detect direction of dis-regulation (up- or down-regulated) in gene sets. The result depends on the type of used ranking metric. When high positive and low negative values of ranking metric represent differentially expressed genes, we can detect gene sets with mostly up-regulated genes or mostly down-regulated genes. When the information about direction of gene expression change due to phenotype is missing, GSEA allows only for detection of dis-regulated gene sets (despite the direction of expression difference). The second case is stated for all four best ranking metrics. These findings are consistent with biological knowledge, where in the same pathway up- and down-regulated genes are observed. Nevertheless, in some biological experiments enrichment of pathways with only up-regulated or only down-regulated genes is desirable, thus while using GSEA researchers have to be aware of its properties.

A common disadvantage of |S2N| and BWS metrics is that a *p*-value for each gene, showing a magnitude of statistical difference in expression between phenotypes, can be only estimated using permutation test [46]. As it is known, when the exact *p*-value is very low, permutation test estimation is very time consuming. By using |MWT| and MSD ranking metrics accurate *p*-values can be obtained without any additional computational cost.

Finally, MSD ranking metric required a higher number of permutations (more than 10,000 in our analysis) to correctly estimate *p*-value for gene set enrichment. This phenomenon is caused by existence of negative values of MSD metric indicating non-significant log fold changes. When we performed label permutation, the variance of gene expression within phenotypes was increased, which lead to huge increase in the number of genes with negative MSD value (and more pathways with negative normalised enrichment score). In GSEA, to calculate a *p*-value for a given pathway only the positive or negative portion of the normalised enrichment score distribution is used, corresponding to the sign of the observed normalised enrichment score. In all cases, when using MSD, the distribution of normalised enrichment score for permuted data consists of a much smaller number of positive values than negative, and thus establishing an adequate *p*-value for positive NES requires more permutations. However, this weakness has also a positive consequence in the form of decreased false positive rate. Furthermore, the MSD was defined in a way that allows implementation even in case of complex linear models or contrasts, thus allowing for a greater flexibility.

## Conclusions

With the use of 28 microarray data sets, contrasting results of gene set enrichment for 16 ranking metrics in GSEA algorithm were observed. All metrics were tested by statistical measures of sensitivity and FPR with the accompanying of computational load. From the group of all tested methods four showed better outcomes, i.e. |MWT|, MSD, |S2N| and BWS. Out of tested metrics the best overall sensitivity was observed for |MWT|, while the best overall FPR estimation was obtained by MSD. In the group of four best metrics, |MWT|, MSD (in current implementation), and |S2N| are based on parametric estimators and should be carefully used when this assumption is not met. BWS shows better outcomes for larger sample size and non-normal gene expression distributions compared to other metrics. It also detects more enriched pathways, keeping false discovery at a reasonable level, which may suggest new discoveries. We showed that choosing ranking metric does matter in case of GSEA and its role is not negligible. Appropriate setting of ranking metric can improve FPR estimation of GSEA method that was criticised in [28]. Nevertheless, it is possible to use any ranking metric, but researchers have to be aware of possible weaknesses presented in this study. In the enclosed MATLAB implementation GSEA computations are efficiently parallelised, giving the opportunity to easily modify the scripts to fulfill researcher expectations. In comparison to existing implementations MrGSEA offers much higher flexibility and functionality in the form of a large scale of ranking metrics (including own one), usage of custom gene set database and two different types of permutation.

## Additional files

Additional file 1 Table with detailed results of surrogate sensitivity, false positive rate and evaluation time for every tested ranking metric on every data set. (XLS 59 kb)

Additional file 2 Table with data sets description about gene expression distribution normality and variance homogeneity. (XLS 38 kb)

## Abbreviations

BWS: Baugmartner-Weiss-Schindler
ccRCC: Clear cell renal cell carcinoma
CI: Confidence interval
FC: Fold change
FCROS: Fold change rank ordering statistics
FCS: Functional class sorting
FPR: False positive rate
GSEA: Gene set enrichment analysis
MrGSEA: MATLAB metric GSEA
MSD: Minimum significant difference
MWT: Moderated welch test
ORA: Over-representation analysis
SoR: Sum of ranks
S2N: Signal-to-noise
WAD: Weighted average difference
